# Distinct Structural Features of the Peroxide Response Regulator from Group A Streptococcus Drive DNA Binding

**DOI:** 10.1371/journal.pone.0089027

**Published:** 2014-02-21

**Authors:** Chang Sheng-Huei Lin, Shi-Yu Chao, Michal Hammel, Jay C. Nix, Hsiao-Ling Tseng, Chih-Cheng Tsou, Chun-Hsien Fei, Huo-Sheng Chiou, U-Ser Jeng, Yee-Shin Lin, Woei-Jer Chuang, Jiunn-Jong Wu, Shuying Wang

**Affiliations:** 1 Department of Microbiology and Immunology, College of Medicine, National Cheng Kung University, Tainan, Taiwan; 2 Physical Biosciences Division, Lawrence Berkeley National Laboratory, Berkeley, California, United States of America; 3 Molecular Biology Consortium, Advanced Light Source, Lawrence Berkeley National Laboratory, Berkeley, California, United States of America; 4 Center of Infectious Disease and Signaling Research, National Cheng Kung University, Tainan, Taiwan; 5 National Synchrotron Radiation Research Center, Hsinchu, Taiwan; 6 Department of Biochemistry and Molecular Biology, College of Medicine, National Cheng Kung University, Tainan, Taiwan; 7 Department of Medical Laboratory Science and Biotechnology, College of Medicine, National Cheng Kung University, Tainan, Taiwan; National Institute for Medical Research, Medical Research Council, London, United Kingdom

## Abstract

Group A streptococcus (GAS, *Streptococcus pyogenes*) is a strict human pathogen that causes severe, invasive diseases. GAS does not produce catalase, but has an ability to resist killing by reactive oxygen species (ROS) through novel mechanisms. The peroxide response regulator (PerR), a member of ferric uptake regulator (Fur) family, plays a key role for GAS to cope with oxidative stress by regulating the expression of multiple genes. Our previous studies have found that expression of an iron-binding protein, Dpr, is under the direct control of PerR. To elucidate the molecular interactions of PerR with its cognate promoter, we have carried out structural studies on PerR and PerR-DNA complex. By combining crystallography and small-angle X-ray scattering (SAXS), we confirmed that the determined PerR crystal structure reflects its conformation in solution. Through mutagenesis and biochemical analysis, we have identified DNA-binding residues suggesting that PerR binds to the *dpr* promoter at the *per* box through a winged-helix motif. Furthermore, we have performed SAXS analysis and resolved the molecular architecture of PerR-DNA complex, in which two 30 bp DNA fragments wrap around two PerR homodimers by interacting with the adjacent positively-charged winged-helix motifs. Overall, we provide structural insights into molecular recognition of DNA by PerR and define the hollow structural arrangement of PerR-30bpDNA complex, which displays a unique topology distinct from currently proposed DNA-binding models for Fur family regulators.

## Introduction

Group A streptococcus (GAS, *Streptococcus pyogenes*) is one of the most aggressive gram-positive human pathogens, responsible for a broad spectrum of diseases with diverse clinical manifestations [1], [2]. Over 500,000 deaths caused by GAS are documented each year around the world [1]. GAS is renowned for its rapidly progressive, highly destructive ability to infect a wide variety of different tissues [3]. GAS infection can develop dramatically from a minor skin lesion to a lethal disease in just a few hours, if left untreated.

Unlike other gram-positive bacteria, GAS does not produce catalase, an oxidoreductase that, in other bacterial species, repairs damage to the bacteria when grown in an aerobic environment. Yet GAS does resist lethal reactive oxygen species (ROS) [4]–[7], through some other means. In addition, the survival and virulence of GAS correlates with the regulation of oxidative stress and metal homeostasis [5], [8], [9]. Therefore, there is considerable interest in how GAS adapts to ROS stress during infection.

PerR is the peroxide response transcriptional regulator that controls genes expression under oxidative stress and it is required for full virulence [5], [9], [10]. PerR belongs to the Fur (ferric uptake regulator) super family known to be dimeric, metal-binding regulators [11]–[14]. PerR has been identified as a repressor that blocks transcription initiation by binding to a specific site (the *per* box) in the promoter region of target genes [5], [15]. One of the targeted genes, *dpr*, containing a putative *per* box sequence in its promoter region, has been found to be directly regulated by PerR in the A-20 GAS strain (M1 serotype) [5], [16]–[18]. Expression of *dpr* in response to a hydrogen peroxide challenge provides protection for bacteria by preventing the Fenton reaction [19], [20]. To understand how *dpr* expression is regulated by PerR, and how PerR interacts with *dpr* promoter DNA, we have conducted a series of mutagenesis, biochemical and structural studies by combining protein crystallography and small-angle X-ray scattering (SAXS). Our results have revealed the PerR-DNA interaction model and illustrated the DNA-binding mode of PerR that is distinct from all other regulators in Fur family.

## Results

### GAS PerR Binds to *dpr* Promoter DNA through *per* Box

**Figure 1:**
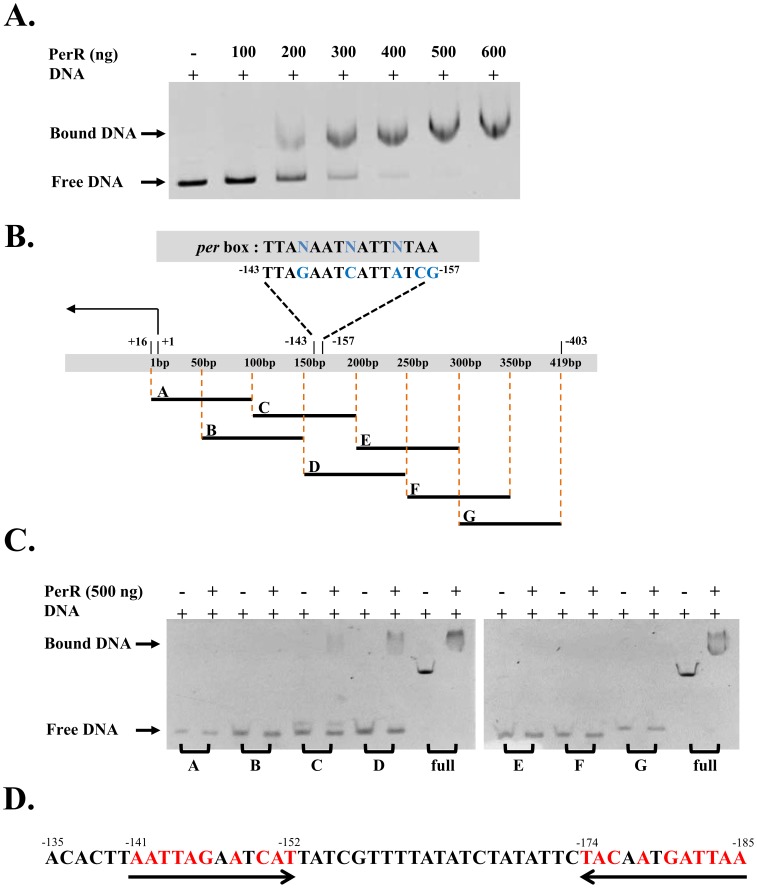
EMSA analysis of PerR DNA-binding ability. (A) The *dpr* promoter from sequence −403 to +16 was incubated with wide-type PerR at the concentrations indicated for 15 min at room temperature. The mixture were separated by 6% native PAGE, and the mobility shift of the promoter was resolved by ethidium bromide staining. (B) Seven DNA fragments were derived from *dpr* promoter. Segments A to G represents sequences as follows: A (−84 to +16), B (−134 to −34), C (−184 to −84), D (−234 to −134), E (−284 to −184), F (−334 to −234) and G (−403 to −284). Putative *per* box was located at sequence between −157 to −143. (C) PerR binding to *dpr* promoter fragments was analyzed by EMSA as described in Figure 1A. (D) PerR binds to *dpr* promoter through sequence −185 to −135. A *per* box similar sequence with opposite direction was found at −185 to −174.

Recombinant 6xHis-tagged PerR protein was purified by Ni-NTA affinity column and size exclusion chromatography to ∼95% purity judged by SDS-PAGE (data not shown). The gel filtration profile of PerR showed that the protein eluted at the volume corresponding to an apparent molecular weight of about 42 kDa (Figure S1). This result is consistent with previous findings that PerR functions as a dimer [21]. PerR binding to the promoter region of *dpr* regulates the gene expression [18]. To confirm that the recombinant PerR protein possesses DNA-binding ability, *dpr* promoter DNA containing 419 bp (−403 to +16) was used to perform an electrophoretic mobility shift assay (EMSA). The PerR protein was able to bind the *dpr* promoter in a concentration dependent manner (Figure 1A), suggesting that the recombinant PerR protein is functional *in* *vitro*. In order to identify the specific binding site of PerR on the *dpr* promoter, seven different PCR-generated ∼100 bp fragments of DNA within the *dpr* promoter at different locations were tested (Figure 1B and 1C). The results showed that PerR binds to the *dpr* promoter from −185 to −135, which includes the previously identified *per* box binding site (from −157 to −143). The DNA-binding sequence includes a region of two 12 bp inverted repeats separated by a 21 bp spacer (Figure 1D). These results demonstrated that the recombinant PerR binds to *dpr* promoter DNA through the *per* box.

### Crystal Structure of PerR

**Figure 2:**
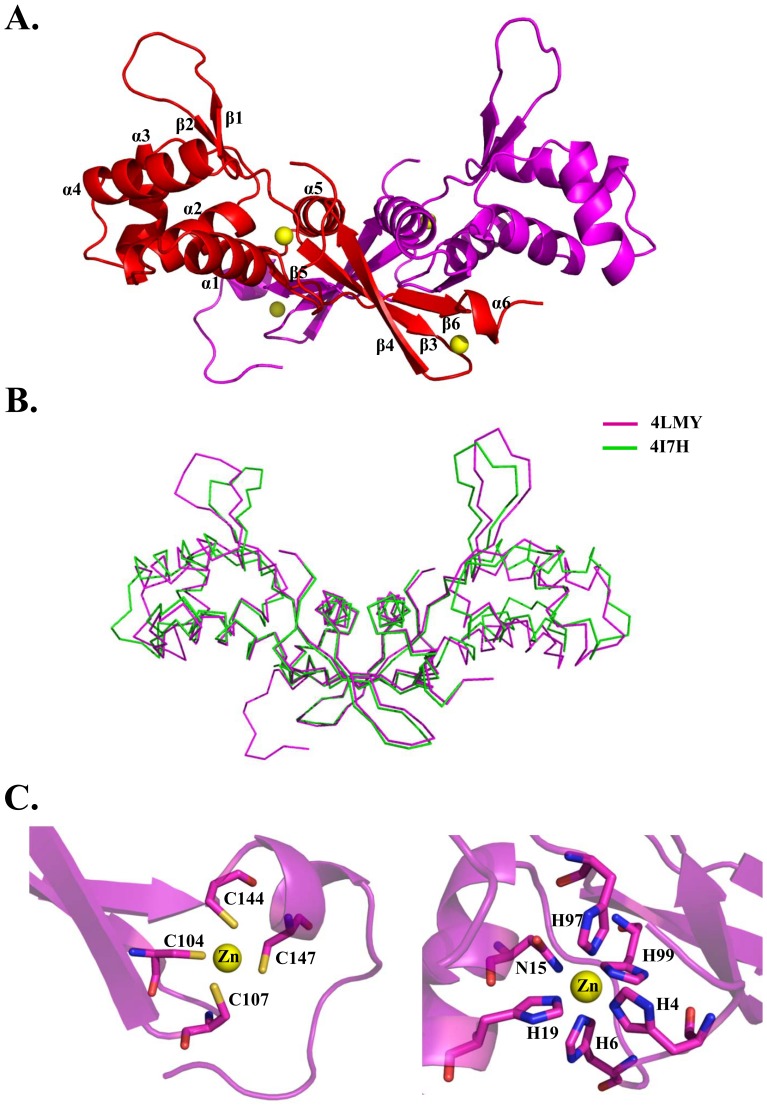
Crystal structure of GAS PerR. (A) Ribbon diagram of the dimeric PerR structure. Each of the monomer is colored in red and magenta. Two zinc metals bound to each PerR subunit are shown as yellow spheres. (B) Superposition of two GAS PerR structures. The PerR-Zn-Zn structure is colored in magenta (PDB code 4LMY); PerR-Zn-Ni is in green (PDB code 4I7H). (C) Magnified view of zinc binding sites on PerR subunit. Left panel, zinc-finger motif coordinated by four cysteine residues (Cys104, Cys107, Cys144 and Cys147). Right panel, zinc-coordinated regulatory site compose of His4, His6, Asn15, His19, His97 and His99 in pseudo octahedral geometry.

We solved the crystal structure of PerR to 1.6 Å resolution by three-wavelength MAD phasing, using zinc as the anomalous scatter [22]. The structure represents the assembly of a homodimer in the asymmetric unit. Each subunit consists of six α-helices (α1 to α6) and six β-strands (β1 to β6) (Figure 2A). Similar to Fur and Fur-like proteins, the GAS PerR monomer can be divided into an N-terminal DNA-binding domain (residues 1–94) and a C-terminal dimerization domain (residues 98–149 in chain A and residues 98–157 in chain B). The two domains are connected by a short linker (residues 95–97) [21], [23], [24]. The X-ray data and refinement statistics are summarized in Table 1. Validation of the structure by program MolProbity [25] showed that no phi-psi angles are in the disallowed regions of the Ramachandran map. The overall structure of PerR resembles the recently published structure (PDB code 4I7H) [21]. Superimposition of two PerR structures for the Cα atoms yields the root mean square deviation of 1.4 Å (Figure 2B).

**Table 1:** Crystallographic data and refinement statistics.

Data collection	PerR: Zn-MAD			Native
	Peak	Inflection	High-Remote	
Wavelength (Å)	1.28276	1.28296	1.25700	1.00000
Space group	*P*2_1_	*P*2_1_	*P*2_1_	*P*2_1_
Cell dimensions (Å)				
*a*	33.33	33.33	33.33	33.11
*b*	87.73	87.73	87.73	87.50
*c*	59.76	59.75	59.76	59.39
*γ*	94.74	94.74	94.74	94.78
Resolution range (Å)	43.86–1.90	43.86–1.90	43.86–1.90	30.0–1.50
	(1.97–1.90)	(1.97–1.90)	(1.97–1.90)	(1.55–1.50)
Completeness (%)	97.6 (91.8)	96.4 (81.6)	97.6 (91.8)	95.6(79.7)
Redundancy	3.79 (3.46)	3.76 (3.28)	3.79 (3.46)	4.8(3.7)
<*I/σI*>	9.2 (2.4)	9.3 (2.7)	9.2 (2.4)	41.8(3.16)
*R*_sym_ a(%)	6.2 (32.9)	6.4 (30.7)	5.9 (33.4)	4.4 (25.1)
Refinement				
Resolution range (Å)				21.2–1.6
*R*_work_ b/*R*_free_ c(%)				22.0/23.9
No. of atoms				
Protein				2459
Ligand/ion				4
Water molecules				185
*B*-factors (Å^2^)				23.66
R.m.s deviations				
Bond lengths (Å)				0.003
Bond angles (°)				0.789

The values in parenthesis are for the highest resolution bin.

a *R*_sym_ = *R*_work_ = ∑_h_∑_i_|I_hi_- <I_h_>|/∑_h_∑_i_I_hi_, were I_hi_ is the *i*th observation of the reflection h, while <I_h_>is the mean intensity of reflection h.

b *R*_factor_ = ∑||F_o_| - |F_c_||/|F_o_|. *R*_free_ was calculated with a small fraction (10%) of randomly selected reflections.

c The *R*_free_ was calculated from 10% of all data that were not used in the refinement.

### C4-type Zn-finger Motif and Zn-bound Regulatory Site

Initially two zinc sites were identified by program SOLVE from the X-ray MAD data and the atoms were located at the C-terminal C-X-X-C zinc finger motif composed by residues Cys104, Cys107, Cys144, and Cys147, in a tetrahedral coordination and consistent with the structure of PDB code 4I7H (Figure 2C, left panel and Figure S2). Interestingly, two peaks of high electron density were apparent during the model building. We performed an X-ray fluorescence emission spectrum and identified the zinc content in the crystals. No other metal emission signals were detected. No exogenous zinc was added during the process of protein purification or crystallization, suggesting that an endogenous zinc is normally bound at the regulatory site, coordinated with residues His4, His6, Asn15, His19, His97, His99 in a pseudo octahedral geometry. The regulatory site of the recently reported PerR structure (PDB code 4I7H) was coordinated by a nickel ion, most likely acquired during Ni-NTA purification [21]. His4 and His6 are located on the loop of the N-terminus, Asn15 and His19 are on the α1 helix, His97 is located on the linker between β2 and β3 β-strands, and His99 is situated on the β3 β-strand (Figure 2C, right panel and Figure S2).

**Figure 3:**
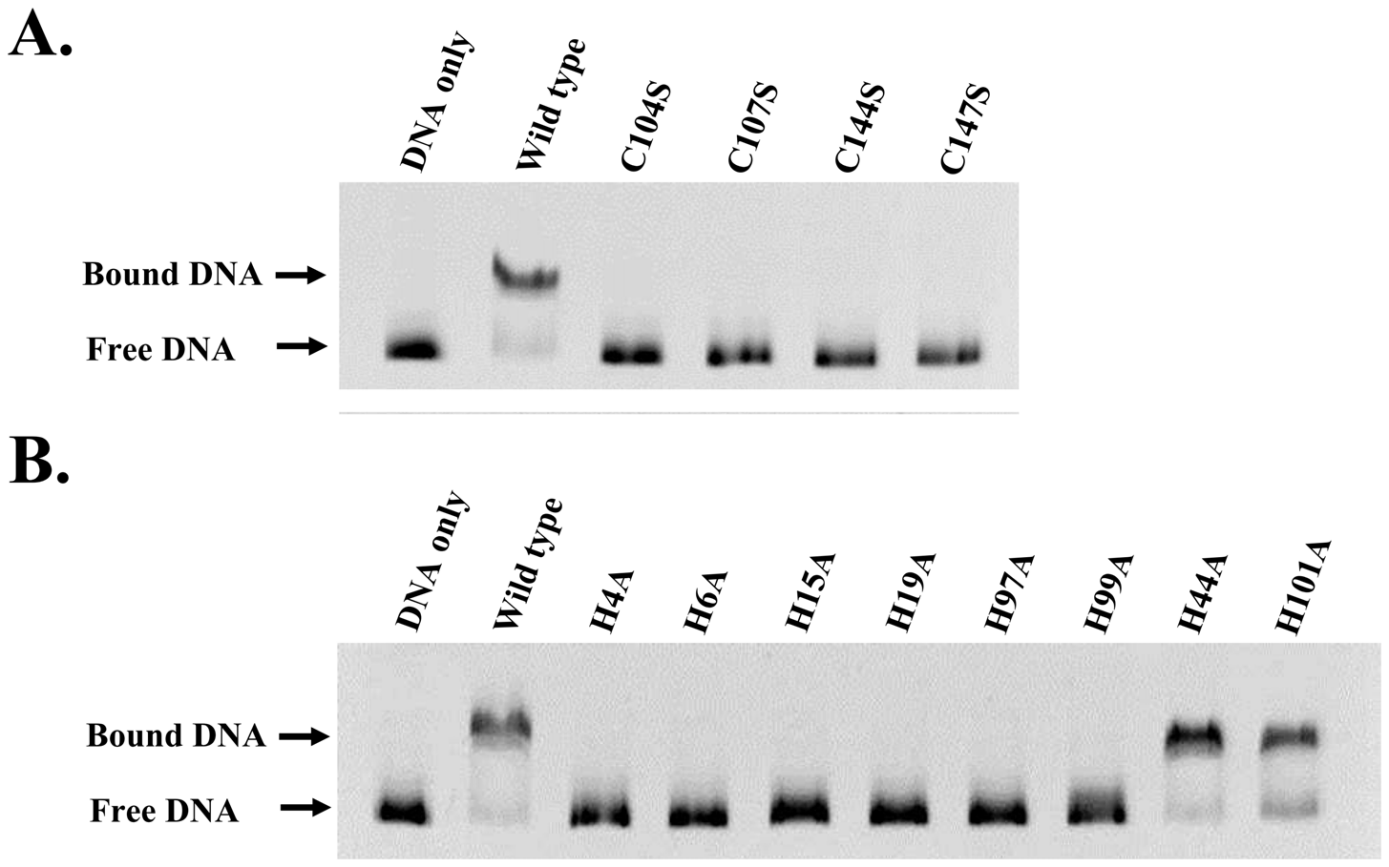
EMSA analysis of PerR mutants. EMSA shows that the residues at regulatory site mutated to alanine abolished the DNA binding of PerR. Mutation of cysteine to serine in the C-terminal zinc-finger motif also abolished the DNA-binding of PerR. H44A and N101A that are not coordinated by zinc are used as controls.

To assess the importance of two metal-binding sites for the DNA-binding activity of PerR, we generated 10 mutants by site-directed mutagenesis. Each residue coordinated by zinc was mutated to alanine or serine: H4A, H6A, N15A, H19A, H97A, H99A, C104S, C107S, C144S, and C147S. Two mutants, H44A and N101A, which are not involving in the metal binding from structural analysis, were used as controls in EMSA. The results showed that mutations in the residues of the zinc-finger motif or of the regulatory site, abolished the DNA binding ability of PerR, but not the control residues H44A and N101A (Figure 3). Therefore, residues involved in metal-binding sites are critical for the DNA binding ability of GAS PerR.

### Solution Structure of PerR by SAXS Studies

**Figure 4:**
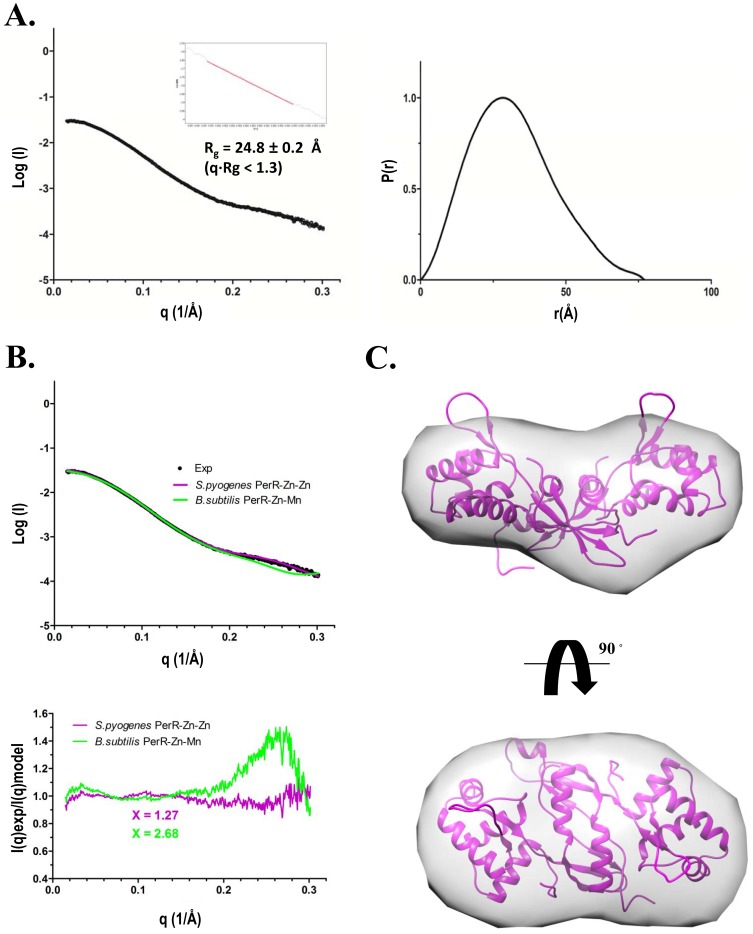
Solution structure of PerR. (A) Experimental scattering profile of GAS PerR was merged from scattering curves of indicated protein concentrations. The R_g_ is derived from linear region of Guinier plot (left panel). Pair-distance distribution function P(r) was computed from program GNOM. The maximal dimension estimated from P(r) is ∼77 Å (right panel). (B) Comparison of experimental scattering curve (black) with theoretical curves calculated from full-atomic models derived from crystal structure of, *S. pyogenes* PerR-Zn-Zn (purple) and, *B. subtilis* PerR-Zn-Mn (green). Residual calculated as I(q)experimental/I(q)model is shown below the scattering curves. (C) Low-resolution SAXS envelope of PerR and the crystallographic PerR structure was fitted into the envelope by the program Chimera [33].

The overall conformation of our GAS PerR-Zn-Zn structure is substantially different from that of the *B. subtilis* PerR-Zn-Mn structure, which displays a DNA-binding-competent conformation [26] (Figure S3). Our recombinant GAS PerR protein with a DNA-binding activity, however, exhibits a DNA-binding-incompetent conformation similar to the *B. subtilis* apo-PerR-Zn structure [24]. Therefore we performed SAXS studies to test whether our crystallographic model agrees with the conformation of PerR in solution. SAXS data were collected at beamline 23A of the National Synchrotron Radiation Research Center, Hsinchu, Taiwan. Scattering curves were collected for purified PerR proteins at various concentrations ranged from 20.0 to 177.6 µM and merged by program PRIMUS [27] (Figure 4A, left panel). From experimental data, values of the radius of gyration (R_g_), were computed from Guinier analysis of low-angle data, and also from the pair-distance distribution function P(r) for individual datasets, to check for consistency. An R_g_ value of 24.8±0.4 Å was calculated from the Guinier region (q•R_g_<1.3) of the scattering curve (Figure 4A, inset in left panel). The best value for D_max_ was ∼77 Å, determined with GNOM [28] by trying a range of values and finding what value gave the best fit to the scattering curve when P(r) was back-transformed (Figure 4A, right panel).

The degree of agreement between crystal structure and solution conformation can be assessed by the χ-value, which measures the goodness-of-fit between the experimental scattering curve and the theoretical scattering profile calculated from structure model by FoXS [29], [30]. Full-atomic modeling is essential for accurate fitting of the experimental data [31], [32], so the missing residues in our PerR structural model were built with the program MODELLER [33]. The theoretically calculated SAXS profile from GAS PerR-Zn-Zn atomistic model fits well with experimental data, with χ = 1.27 (Figure 4B). The *B. subtilis* PerR-Zn-Mn atomistic model showed less well fit to GAS PerR experimental data at scattering angle q ranged from 0.2 to 0.3 Å^−1^ (corresponding to ∼30–20 Å resolution), consistent with the conformational disparity between two crystal structures at N-terminus (Figure S3). To model the low-resolution solution structure, molecular shapes were computed and averaged with the program DAMMIN [34] and DAMAVER [35]. The PerR crystal structure was superimposed onto the molecular SAXS envelope by program SUPCOMB [36] (Figure 4C). SAXS studies show that the crystallographic structure of GAS PerR is indeed consistent with its solution formation. All experimental parameters from SAXS data are presented in Table 2 according to the publication guideline of small-angle scattering data [37].

**Table 2:** SAXS data collection and statistics.

	PerR	PerR-DNA mixture		
		1∶1	2∶1	4∶1
**Data-collection parameters**				
Instrument	Synchrotron 23A SWAXS endstation of NSRRC			
Beam geometry	0.5 mm dia. beam			
Wavelength (Å)	0.8857	0.82825	0.82825	0.82825
*q* range (Å^−1^)	0.0085–0.37	0.007–0.39	0.007–0.39	0.007–0.39
Exposure time (s)	300^a^	300^a^	300^a^	300^a^
Concentration range (µM)	20.0	Protein 26.5	Protein 26.5	Protein 26.5
	68.1	DNA 26.5	DNA 13.3	DNA 6.63
	177.6			
Temperature (K)	288	288	288	288
**Structural parameters**				
*R_g_* (Å) [from P(r)]	24.58±0.04	39.11±0.04	38.62±0.06	36.59±0.08
*R_g_* (Å) [from Guinier]	24.8±0.4	38.9±0.3	38.1±0.1	35.5±0.2
*I(0)* (cm^−1^) [from Guinier]	0.033	0.12	0.08	0.05
	0.113			
	0.352			
*D*_max_ (Å)	∼77	∼125	∼125	∼125
Porod volume estimate (Å^3^)	∼58100^b^	∼354000	∼335000	∼122000
Dry volume calculated from sequence (Å^3^)	37682	N/A	N/A	N/A
**Molecular-mass determination**				
Partial specific volume (cm^3^g^−1^)	0.73	N/A	N/A	N/A
Contrast (Δρ*10^−10^cm^−2^)	3.148	N/A	N/A	N/A
Molecular mass *Mr*	∼35	∼80	∼50	∼40
[estimated from Porod Volume] kDa				
Calculated monomeric *M_r_* from sequence	37700	N/A	N/A	N/A
**Software employed**				
Primary data reduction	NSRRC 23A SWAXS package			
Data processing	SCATTER 1.7 and ATSAS 2.4			
*Ab initio* analysis	DAMMIN	N/A	N/A	N/A
Validation and averaging	DAMAVER	N/A	N/A	N/A
Rigid-body modelling	N/A	N/A	N/A	N/A
Computation of model intensities	FoXS	FoXS	FoXS	FoXS
Envelope representations	Chimera	N/A	N/A	N/A
Minimal ensemble analysis	N/A	MES	MES	MES

*a*. 300s with 60s single exposure time for 5 successive exposures.

*b*. Only globular proteins with smooth surfaces: “Porod volume” ∼ “Dry volume”; uncertainty is contributed mainly from Q-invariant (upper limit of 20% is used with NSRRC 23A SWAXS)”.

### DNA-binding Residues of PerR

**Figure 5:**
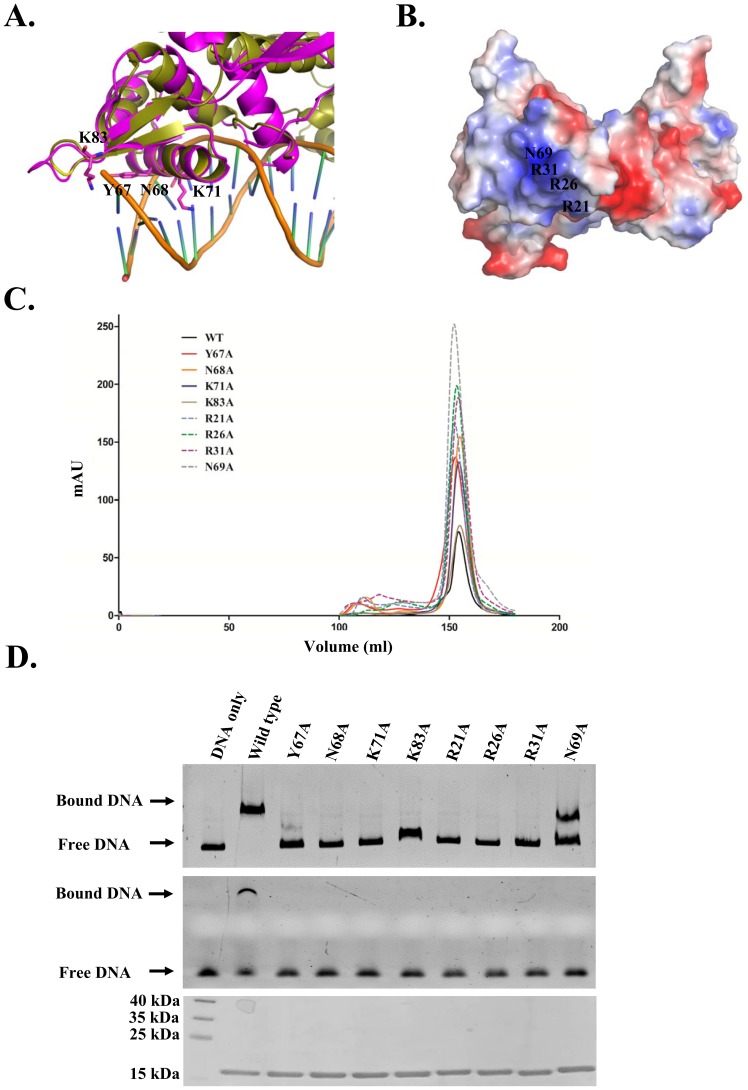
Analysis of PerR DNA-binding residues. (A) Superposition of the winged-helix motif of *S. aureus* BlaI-DNA complex (PDB code 1XSD) with GAS PerR. Yellow, *S. aureus* BlaI-DNA complex; Magenta, GAS PerR. Predicted residues (Y67, N68, K71 and K83) related to DNA-binding are labeled and shown as sticks. (B) Surface electrostatic potential representation of PerR. Residues (R21, R26, R31 and N69) with strong positive charge are labeled. (C) Size-exclusion chromatographic profile of PerR (black) and PerR mutants (red, Y67A; orange N68A; blue, K71A; brown, K83A; blue dashed line, R21A; green dashed line, R26A; purple dashed line, R31A; grey dashed line, N69A). (D) EMSA analysis of binding activity of the mutant PerR proteins to *dpr* promoter (−403 to +16, top panel) and *dpr* segment (−185 to −135, middle panel). A coomassie blue stained SDS-PAGE with equal amounts of wide-type or mutant PerR is shown below EMSA gels.

Our PerR structure contains an N-terminal winged-helix motif consisting of α2-α4 and β1-β2. To identify the potential residues involved in the contact interface between PerR and *dpr* promoter, we superimposed the winged-helix motif of PerR with that of the *Staphylococcus aureus* BlaI-DNA complex structure (PDB code 1XSD) [38]. This study suggests that four residues in PerR Tyr67, Asn68, Lys71 and Lys83 might be involved in the interaction with DNA (Figure 5A). The surface electrostatic potential of PerR contains a strong positively-charged patch including residues Arg21, Arg26, Arg31 and Asn69 (Figure 5B). To determine whether these residues were involved in DNA interaction, each residue was substituted with an alanine. Mutants were constructed and purified up to 95% purity. All the gel-filtration profiles were consistent with wild-type PerR, suggesting that no mutation affected the protein dimerization (Figure 5C). The mobility shift assay showed that alanine substitution of any of R21A, R26A, R31A, N68A or K71A abolished DNA-binding by PerR. Alanine substitution of any of Y67A, N69A and K83A also reduced but did not completely eliminate DNA binding (Figure 5D). This study strongly suggests that residues Arg21, Arg26, Arg31, Asn69, Tyr67, Asn68, Lys71 and Lys83, located on the positively charged patch, are involved in PerR interaction with DNA.

### Structural Characterization of the PerR-DNA Complex by SAXS Analysis

**Figure 6:**
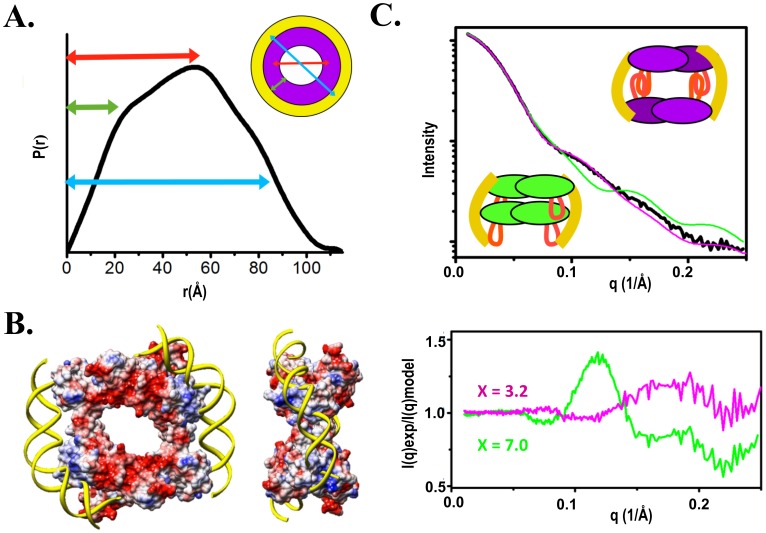
Molecular architecture of PerR-DNA complex. (A) The P(r) function calculated from experimental SAXS profile of a PerR:DNA 1∶1 complex indicates multilayers-hollow structural assembly. (B) Two PerR homodimers satisfy this hollow assembly. Surface electrostatic potential representation of assembled PerR tetramer indicates strong positive charges (blue) where the 30 bp DNA was manually docked. (C) The theoretical SAXS curve calculated from the built PerR-30bpDNA complex showed good fit (magenta) to the experimental SAXS data (black) (χ = 3.2). An alternative complex model, with a compact assembly in which the head-to-tail PerR tetramer was wrapped by DNA, showed poor fit to the experimental data (χ = 7.0) (green).

Currently, no structural information is available for the PerR-DNA complex. In order to resolve its molecular architecture, we have performed SAXS studies. To explore the stoichiometry of PerR-DNA complex formed in solution and to investigate if the stoichiometry varies when mixing different ratios of PerR and DNA, we collected SAXS data on samples containing mixtures of 1∶1, 2∶1, and 4∶1 PerR:DNA ratios (Table 2 and Figure S4). The DNA is a 30 bp fragment of the *dpr* promoter ranging from −135 to −164 covering the *per* box. P(r) function for 1∶1 PerR:DNA ratio with the maxima at the ∼60 Å and maximal dimension ∼100 Å strongly indicate hollow structural arrangement of PerR-DNA assembly (Figure 6A). Additionally, P(r) with a shoulder at the ∼80 Å indicates additional density layer surrounding overall ring-like conformations (Figure 6A). Based on the information from P(r), mutagenesis studies, and surface electrostatic potential of PerR, we manually built an initial model of the PerR-DNA complex. The model consists of two PerR homodimers which are held together by two 30 bp DNA fragments interacting with the positively-charged patches from two adjacent dimers (Figure 6B). Formation of the PerR tetramer-DNA complex was also confirmed by Porod Volume calculation indicating ∼80 kDa large assembly for 1∶1, ∼50 kDa for 2∶1, and ∼40 kDa for 4∶1 PerR:DNA ratios (Table 2) [39]. The proposed head-to-head tetrameric assembly of PerR in the model satisfies hollow assembly as indicate by P(r) shape and P(r) shoulders (Figure 6A and 6B). The theoretical SAXS curve calculated from this PerR-30bpDNA model by program FoXS [29], [30] showed good fit to the experimental SAXS data (χ = 3.2) (Figure 6C and Figure S5A). An alternative PerR-30bpDNA model with a compact assembly of PerR tetramer in head-to-tail arrangement showed poor fit to the experimental data (χ = 7.0) (Figure 6C). SAXS data of samples with 2∶1 and 4∶1 PerR:DNA ratios show the same Dmax ∼ 125 Å (Figure S4D), suggesting the complex formed from different mixtures is the same as the proposed PerR-30bpDNA model from 1∶1 PerR:DNA ratio. To understand the dynamic assembly of PerR-DNA complex, Minimal Ensemble Search (MES) [40] was applied to define the population of the dissociated species from the proposed model. Four different models (unbound DNA, protein, partial complex and complex) were input into MES for selecting the ensemble to optimize the fit to the experimental SAXS profile (Figure S5). The best ensemble was calculated to best fit the experimental curve by minimizing the discrepancy χ between the experimental and calculated profile. The scattering from such a minimal ensemble is computed by weighting the individual scattering profile from selected state. For 1∶1 PerR:DNA ratio, the fit was improved (χ = 3.2 to 2.5) when the ensemble of 96% PerR-DNA complex and 4% PerR dimers was selected (Figure S5A). For 2∶1 and 4∶1 PerR:DNA ratios, the selected ensembles significantly improved the fits than the single PerR-DNA complex model (Figures S5B and S5C). The excess of protein in the mixtures results in dissociate products of free protein and partial complex assembly, but does not form the higher-order PerR-DNA complex. Taken together, SAXS results allowed us to define the molecular assembly of PerR with a sequence-specific 30 bp DNA.

## Discussion

PerR is a peroxide sensor and plays a crucial role in GAS oxidative resistant response. The biological functions of PerR in peroxide sensing, metal binding, and gene regulation have been well studied. However, the molecular mechanism by which PerR recognizes DNA has not yet been clearly elucidated due to the lack of structural information of PerR-DNA complex. In this work, we provide the first structural insight for PerR-DNA complex and present a model which illustrates the physical association of PerR with its cognate promoter DNA.

Our high-resolution PerR crystal structure revealed that an endogenous zinc is bound at the regulatory site (Figure 2), unlike what was found in two other PerR crystal structures [21], [26]. Zinc ion is the second most common transition metal in the human body, playing important roles in the host immune system [41], [42]. Zn(II) is also an essential nutrient for bacteria, but exhibits toxicity at high concentrations. PerR regulates several zinc homeostasis-related genes, such as *pmtA* [8], *adcR* and *adcB* [43]. A PerR-deficient mutant shows hyper-resistant to zinc toxicity [8]. AdcRCB (*SPy0092*-*SPy0094*) was identified as a transport complex for streptococcal Mn^2+^/Zn^2+^ import [44], [45]. PerR-dependent expression of AdcR regulon is important in metal homeostasis [43]. Collective information suggests that GAS uses zinc as a signal to activate the PerR regulon to cope with toxicity from high zinc concentrations.

GAS PerR exhibits different dimeric conformation compared to the DNA-binding-competent conformation of the *B. subtilis* PerR-Zn-Mn (Figure S3), suggesting an alternative DNA-binding mode. To exclude the conformational difference is not due to the crystallographic artifact, we performed SAXS studies which confirmed the crystal structure indeed reflects the PerR solution conformation (Figure 4).

A recently published paper indicated that H44 is important in *pmt*A promoter DNA binding [21]. However, our EMSA analysis showed that mutation of H44A did not affect *dpr* binding ability of PerR. To define the DNA-binding interface of PerR, we constructed eight alanine-substituted mutants and confirmed that residues on the winged-helix motif and the positive surface patch are involved in DNA recognition. H44 is distant from our identified DNA-binding residues, consistent with the EMSA results that H44 is not involved in the *dpr* promoter DNA recognition (Figure 3B).

Besides PerR, the Fur family includes iron sensor Fur [46], zinc sensor Zur [47], [48], nickel sensor Nur [49] and manganese sensor Mur [50], which are all structurally similar [23], [51], [52]. How these regulators with structural resemblance recognize divergent DNA sequences is a long-standing fundamental question. Several protein-DNA complex models were proposed for the Fur family, suggesting that regulators interact with DNA by N-terminal helix-turn-helix/winged-helix in a DNA-binding-competent homodimeric conformation [53], [52], [21]. Here, we describe a PerR-30bpDNA complex model and delineate the hollow structural architecture with PerR tetramer wrapped by two DNA fragments (Figure 6). In the model, the DNA-binding interface is contributed from two adjacent PerR homodimers in tetrameric assembly. This distinct DNA-binding feature explains that PerR dimer alone is in the DNA-binding-incompetent conformation. In conclusion, this study confers insights into the molecular basis of DNA recognition by PerR and offers a structural framework of PerR-DNA complex for Fur family.

## Materials and Methods

### Protein over Expression and Purification

The full-length PerR of GAS serotype M1 A20 was cloned into a pET-21b vector between *NdeI* and *XhoI* restriction sites and transformed into *E. coli* BL21 (DE3). Expression of the recombinant 6xHIS-tagged PerR protein was induced by adding 0.5 mM IPTG (isopropyl-β-D-thiogalactopyranoside) to the culture when cells reached an O.D._600_ of 0.6, and further incubated at 25°C for 4 hr. Cells were centrifuged at 8,000 rpm for 20 min, resuspended in buffer A (20 mM Tris-HCl, 200 mM NaCl, pH 7.5) and disrupted by sonication on ice. Supernatant was loaded into a Ni-NTA column (GE Healthcare) and unbound proteins were washed away with 60 mM imidazole in buffer A. PerR proteins were eluted with 300 mM imidazole in buffer A. Fractions containing PerR proteins were pooled and further purified by Superdex™ 75 size exclusion chromatography (GE Healthcare). Subsequently, proteins were dialyzed in buffer B (20 mM Tris-HCl, 150 mM NaCl, 0.5 mM EDTA, 5% glycerol, and 3 mM DTT, pH 7.5). Purified proteins were stored at 4°C for further use. The PerR mutant plasmids were constructed by the method of *Dpn* I-dependent site-directly mutagenesis (SDM) or overlap extension PCR (OE-PCR) using wild-type PerR as a template. All primers used for construction of PerR and PerR mutant plasmids are listed in Table S1. Overexpression and purification of PerR mutant proteins were performed as described above.

### Electrophoretic Mobility Shift Assay (EMSA)

PerR binding to promoter DNA containing a putative PerR-binding sequence was performed as previously described [18]. Various segments of *dpr* promoter DNA were amplified by PCR using wild-type A20 as a template (Table S2). Purified PerR and promoter DNA were incubated in 20 µl binding buffer (20 mM Tris pH 8.0, 50 mM KCl, 5% glycerol, and 50 µg/ml bovine serum albumin) for 15 min at 25°C. The mixtures were separated by 6% native polyacrylamide gels running with Tris-borate buffer (45 mM Tris-base, 45 mM boric acid). The gels were stained with ethidium bromide and visualized by a UV imaging system.

### Crystallization

Purified PerR proteins were concentrated to 8–10 mg/ml in buffer B for crystallization trials. Various commercial kits of Hampton Research and Emeralds BioSystems were initially screened for crystallization conditions of the PerR protein, by the vapor diffusion method, performed with a Honeybee 961 robot (Digilab Genomic Solutions). PerR crystals were obtained in sitting drops containing 0.5 µl of protein and 0.5 µl of various precipitation solutions at 25°C within three days. Diffraction-quality crystals were obtained by the hanging drop diffusion method mixing 2 µl of protein and 2 µl of solution (0.1 M Tris-HCl pH 8.6, 0.25 M MgCl_2_, and 27% PEG 4000). Crystals were flash-frozen using 30% PEG 4000 as a cryoprotectant in a stream of nitrogen gas at ∼100 K prior to data collection.

### X-ray Data Collection and Processing

Native diffraction data were collected to 1.5 Å resolution at beamline BL13B1 equipped with ADSC Q315 CCD detector at National Synchrotron Radiation Research Center (NSRRC) in Hsinchu, Taiwan. 250 frames were collected; each with 1° oscillation and exposed for 2 sec at the wavelength of 1.0 Å with the crystal-to-detector distance of 200 mm at the temperature 100 K. Diffraction data were processed by HKL2000 program [54].

The Zn multi-wavelength anomalous (Zn-MAD) data sets were collected at beamline 4.2.2 equipped with a NOIR-1 detector at the Advanced Light Source (ALS) of the Lawrence Berkeley National Laboratory, Berkeley, USA. A three-wavelength MAD dataset was collected to 1.8 Å resolution at wavelengths of 1.28276 Å, 1.28296 Å, and 1.25700 Å, corresponding to Zn absorption peak, edge, and remote high energy. 360 frames were collected for each wavelength; each frame was exposed for 1 sec with the distance of 100 mm from crystal to detector. Diffraction data were processed with d^*^TREK [55]. Crystal parameters and data collection statistics are summarized in Table 1.

### Crystal Structure Determination and Refinement

Zinc sites were determined from anomalous difference Patterson maps computed from the MAD data using the automated search algorithms of the program SOLVE [56]; two zinc sites were initially identified in the asymmetric unit. MAD phases gave an initial overall figure of merit of 0.45 for all data to 2.5 Å. The experimental electron density map was improved by density modification with the program RESOLVE [56]. Subsequent model building was carried out with the program Coot [57]. A peak of high electron density in each monomer was recognized as a metal ion in the structure and confirmed to be zinc by crystal X-ray fluorescence (described below). Multiple cycles of simulated annealing, positional, and individual isotropic B factor refinements against native data to 1.6 Å were performed with REFMAC [58] and PHENIX [25], alternating with manual model rebuilding in Coot [57]. A total of 185 water molecules were included in the final model and in the final stage of refinement. The refinement statistics are summarized in Table 1. All structure representation figures are generated by the program PyMOL (http://www.pymol.org).

### Crystal X-ray Fluorescence

At beamline 4.2.2 of ALS, X-ray fluorescence from a single crystal was excited with radiation of energy 11,000 eV, and the fluorescence emission spectrum from the crystal was recorded with a silicon drift diode coupled to a multichannel analyzer. Peaks at the energies expected for the K_α_ (8630 eV) and K_β_ (9572 eV) emissions of zinc were observed, at the appropriate amplitude ratios. No other significant emission signals were seen.

### SAXS Data Collection

Data were collected at the NSRRC SWAXS beamline 23A, Hsinchu, Taiwan. PerR protein samples and PerR-DNA complexes were dialyzed against corresponding buffers prior to data collection. At beamline 23A, 60 µl of sample were loaded into thermostatic cells at 15°C. With 14 keV X-rays (wavelength ≤ = 0.8857 Å) and a sample-to-detector distance of 2.5 m, the scattering wave vector *q*, defined by 4πλ^−1^sinθ with scattering angle 2θ, is covered from 0.0085 Å^−1^ to 0.37 Å^−1^. Data were collected with 1 frame per 60 s for 5 successive frames using a Pilatus 1M-F detector; frames were selectively combined for data reduction. Sample solution scattering was subtracted by the corresponding buffer solution scattering collected under identical experimental conditions, and was scaled to the absolute intensity scales [59].

### SAXS Data Analysis and Modeling

Following data reduction, data were analyzed with the programs ATSAS 2.4 [60] and SCATTER. The values of radius of gyration (R_g_) were first computed from Guinier plots [61] to test for aggregation. Scattering curves of PerR measured at different protein concentrations were merged using the program PRIMUS [27] and SCATTER. For PerR and PerR-DNA complex, distance distribution functions P(r) were computed as the Fourier transform of the scattering profiles using the program GNOM [28] and SCATTER. Theoretical SAXS profiles were calculated using program FoXS [29], [30]. SAXS envelopes of PerR were computed with the program DAMMIN [34]. Ten runs were performed to verify the stability of the solution and the envelopes were averaged by DAMAVER [35]. Atomistic model of PerR-30bpDNA complex was built by combining two PerR homodimer to satisfy hollow assembly as indicated by P(r) shape (Figure 6) and P(r) shoulders. Minimal Ensemble Search (MES) [40] was applied to select the assembly of the mixed states for the best fit to experimental data.

### PDB Accession Code

Coordinates and structure factors have been deposited in the Protein Data Bank with the identifier 4LMY.

## Supporting Information

Figure S1 **Size-exclusion chromatographic profile of the recombinant 6xHis-tagged PerR protein.** Elution volumes of standard molecular weight markers (black) and purified 6xHis-tagged PerR (red).
(TIF)

Figure S2 **Zinc finger motif and regulatory site of PerR structure.** Superposition of GAS PerR-Zn-Zn (magenta; PDB code 4LMY) and PerR-Zn-Ni (green; PDB code 4I7H) at zinc-finger motif (left) and metal-bound regulatory site (right). Zn/Ni ion is colored in yellow.
(TIF)

Figure S3 **Crystal structures of GAS PerR-Zn-Zn and** ***B. subtilis*** **PerR-Zn-Mn.** Superposition of GAS PerR-Zn-Zn (magenta; PDB code 4LMY) and *B. subtilis* PerR-Zn-Mn (cyan; PDB code 3F8N).
(TIF)

Figure S4 **Small-angle X-ray scattering data of the PerR-DNA complex.** Experimental scattering profiles of (A) 1∶1, (B) 2∶1, and (C) 4∶1, PerR:DNA ratio. Insets show the Guinier plot with linear fit and the corresponding radius of gyration (R_g_) values. (D) P(r) functions of 1∶1 (red), 2∶1 (blue), and 4∶1 (green), PerR:DNA ratios. P(r) functions are normalized to unity of their maxima. The maximal distance estimated from P(r) functions is ∼125 Å for all ratios. Distinct disappearing of r ∼60 Å P(r) maxima for the 2∶1 and 4∶1 ratios indicates dynamic protein-DNA assembly as shown in the Figure S5.
(TIF)

Figure S5 **Minimal ensemble analysis of PerR-DNA complex mixtures.** Ensemble fit (blue) versus PerR-30bpDNA model fit (red) by FoXS-MES for (A) 1∶1, (B) 2∶1, and (C) 4∶1, PerR:DNA ratio. Residual calculated as I(q)experimental/I(q)model is shown below the scattering curves. Chi value (χ) versus ensemble size is shown in histogram. The selected ensemble size from four input species (unbound DNA, protein, partial complex, complex) for each PerR:DNA ratio by MES is colored in blue in histogram and the selected species are represented in cartoon.
(TIF)

Table S1 **Primers for plasmids construction.**
(DOC)

Table S2 **Primers for PCR-generated** ***dpr*** **promoter DNA fragments.**
(DOC)
