# Supplementary figures and images for: Distinct Structural Features of the Peroxide Response Regulator from Group A Streptococcus Drive DNA Binding
Source: PLoS One. 2014 Feb 21;9(2):e89027. doi: 10.1371/journal.pone.0089027 (PMC3931707; doi:10.1371/journal.pone.0089027)

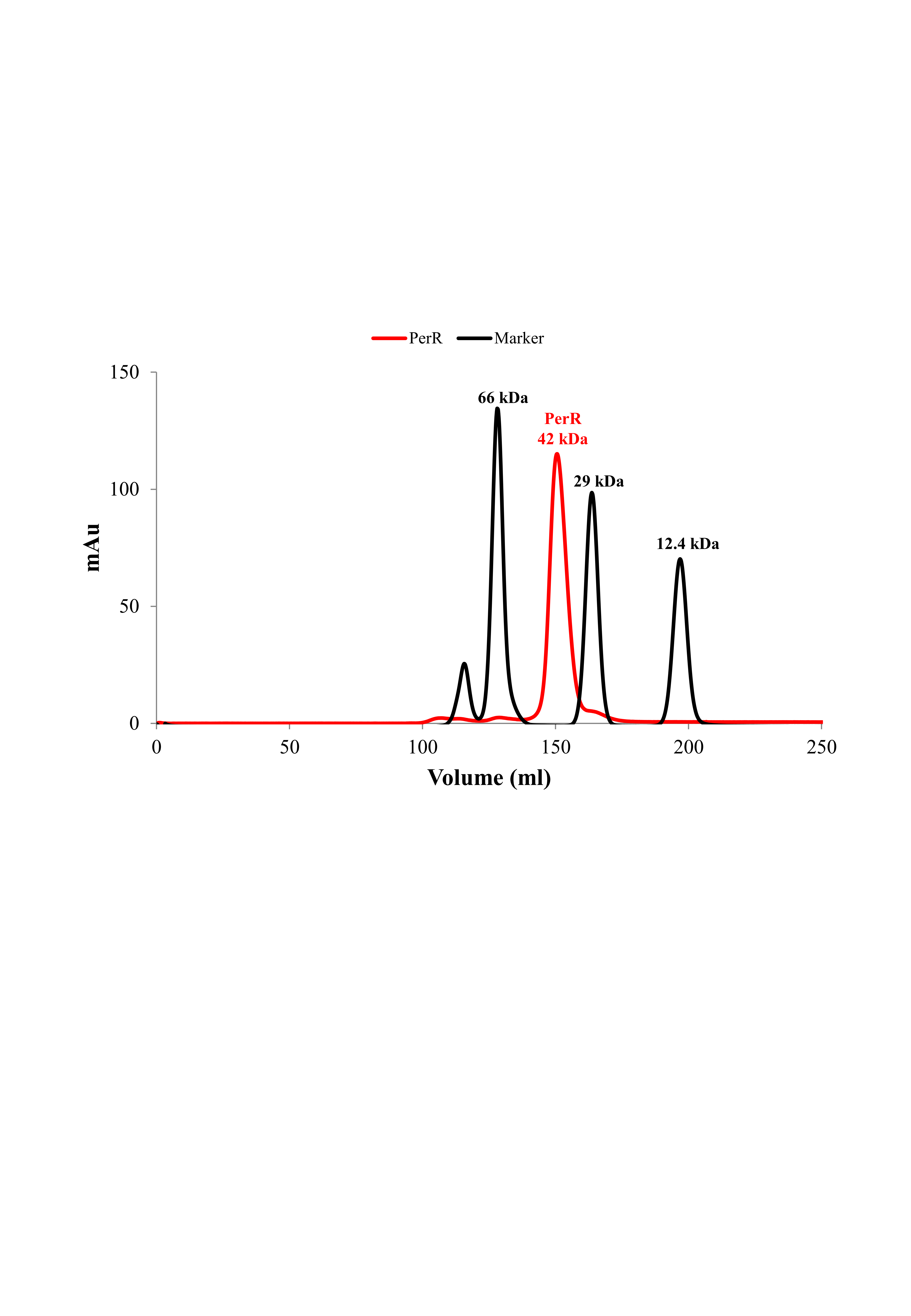

Supplement: Figure S1 — Size-exclusion chromatographic profile of the recombinant 6xHis-tagged PerR protein. Elution volumes of standard molecular weight markers (black) and purified 6xHis-tagged PerR (red). (TIF) [file pone.0089027.s001.tif]

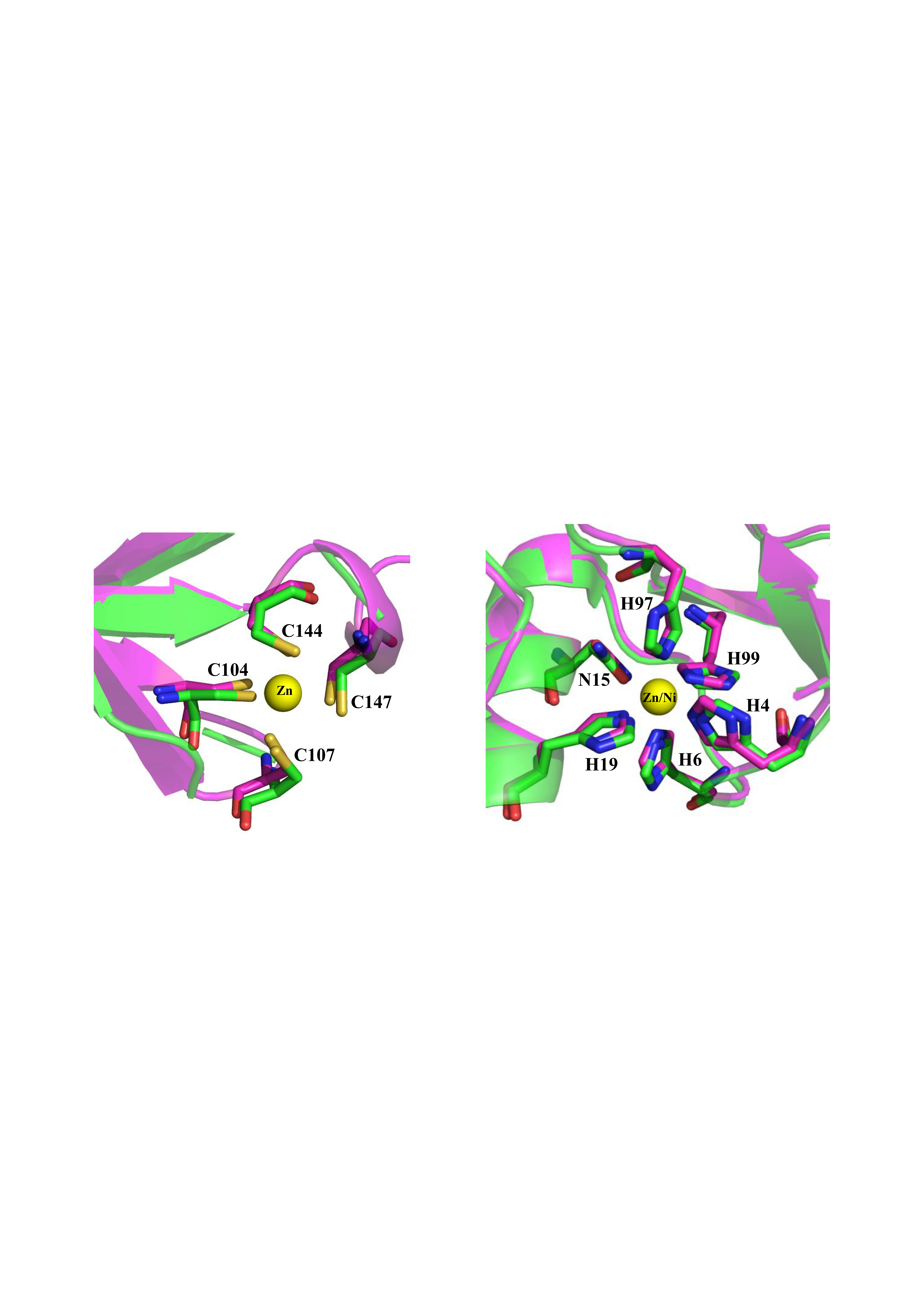

Supplement: Figure S2 — Zinc finger motif and regulatory site of PerR structure. Superposition of GAS PerR-Zn-Zn (magenta; PDB code 4LMY) and PerR-Zn-Ni (green; PDB code 4I7H) at zinc-finger motif (left) and metal-bound regulatory site (right). Zn/Ni ion is colored in yellow. (TIF) [file pone.0089027.s002.tif]

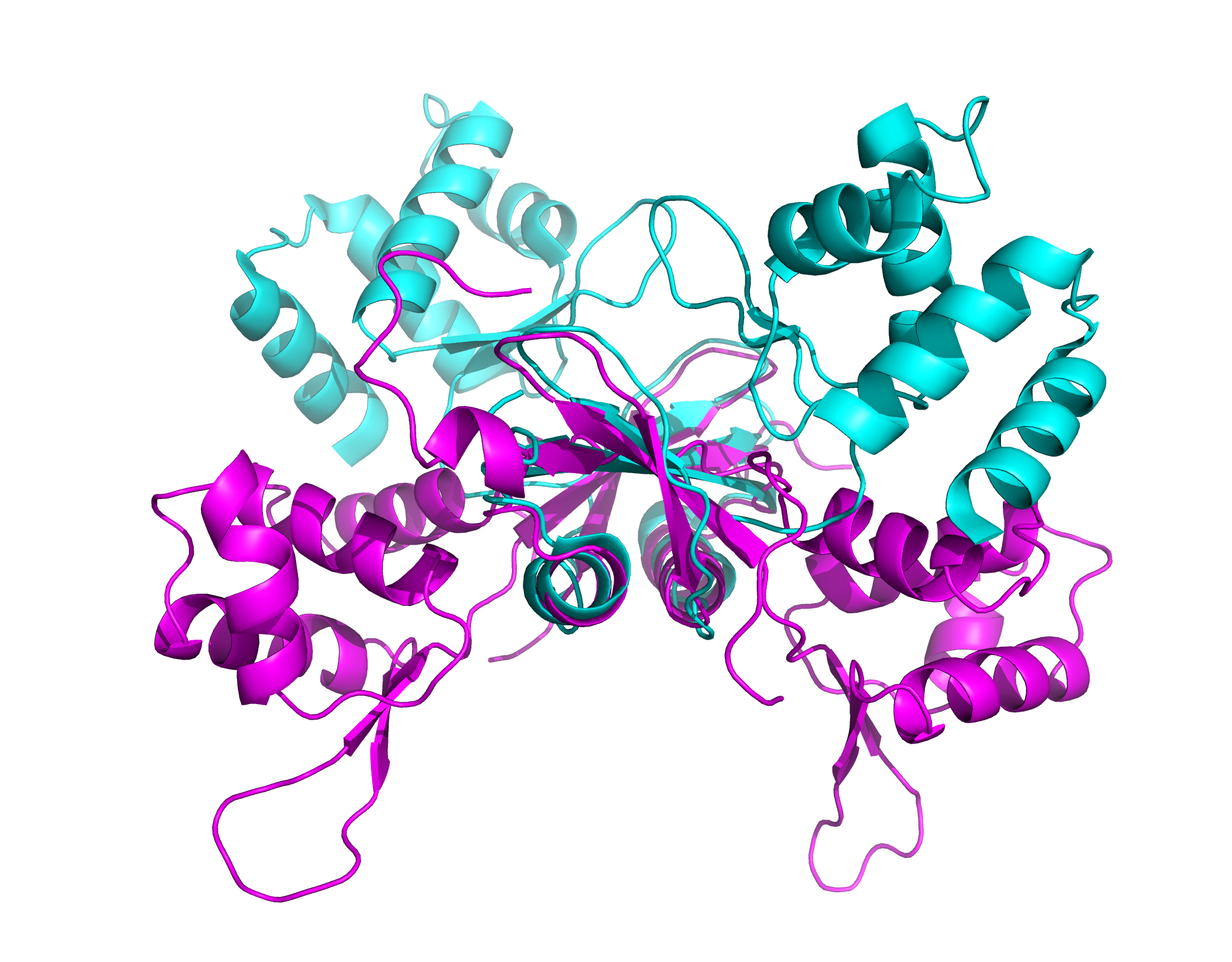

Supplement: Figure S3 — Crystal structures of GAS PerR-Zn-Zn and B. subtilis PerR-Zn-Mn. Superposition of GAS PerR-Zn-Zn (magenta; PDB code 4LMY) and B. subtilis PerR-Zn-Mn (cyan; PDB code 3F8N). (TIF) [file pone.0089027.s003.tif]

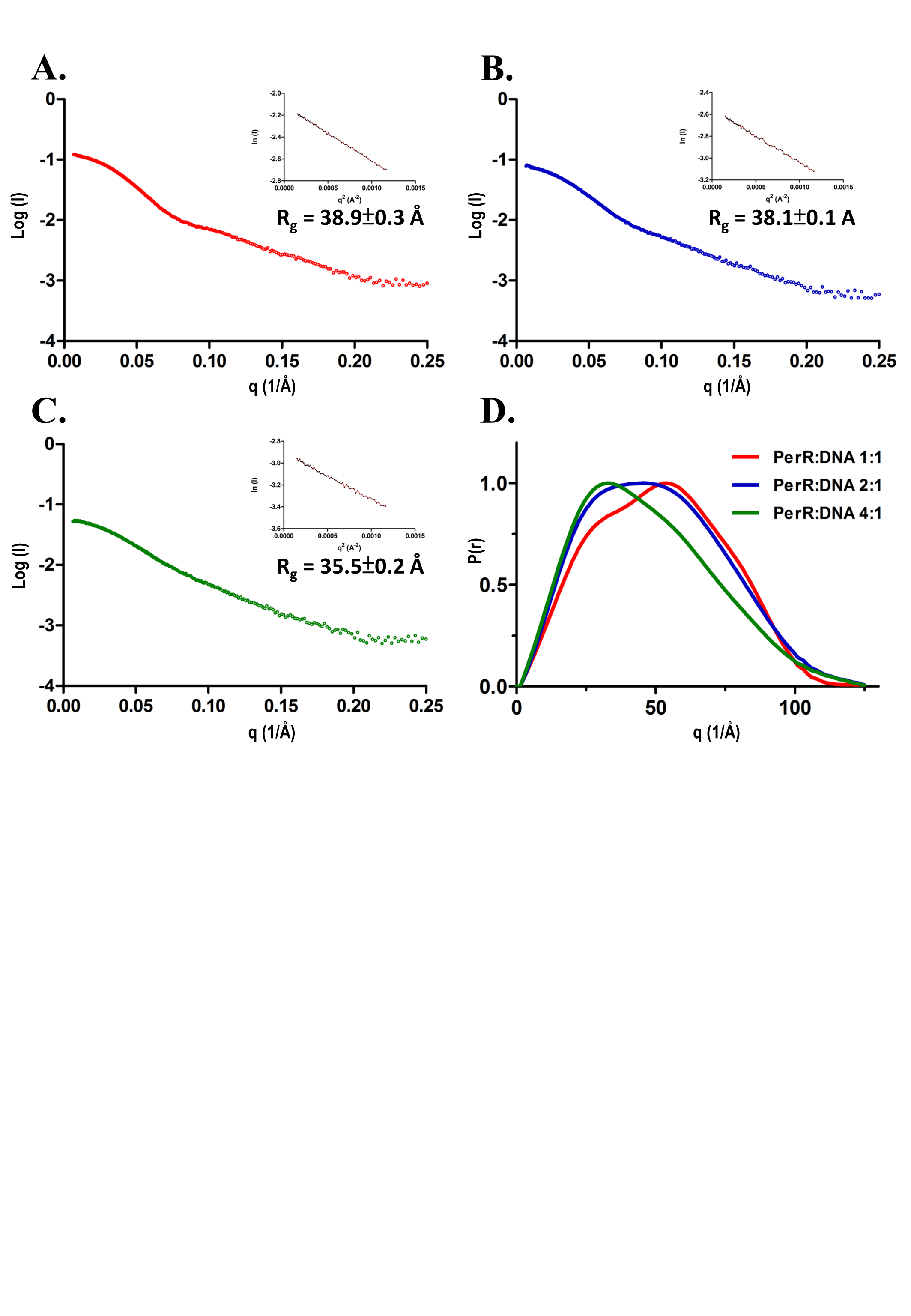

Supplement: Figure S4 — Small-angle X-ray scattering data of the PerR-DNA complex. Experimental scattering profiles of (A) 1∶1, (B) 2∶1, and (C) 4∶1, PerR:DNA ratio. Insets show the Guinier plot with linear fit and the corresponding radius of gyration (Rg) values. (D) P(r) functions of 1∶1 (red), 2∶1 (blue), and 4∶1 (green), PerR:DNA ratios. P(r) functions are normalized to unity of their maxima. The maximal distance estimated from P(r) functions is ∼125 Å for all ratios. Distinct disappearing of r ∼60 Å P(r) maxima for the 2∶1 and 4∶1 ratios indicates dynamic protein-DNA assembly as shown in the Figure S5. (TIF) [file pone.0089027.s004.tif]

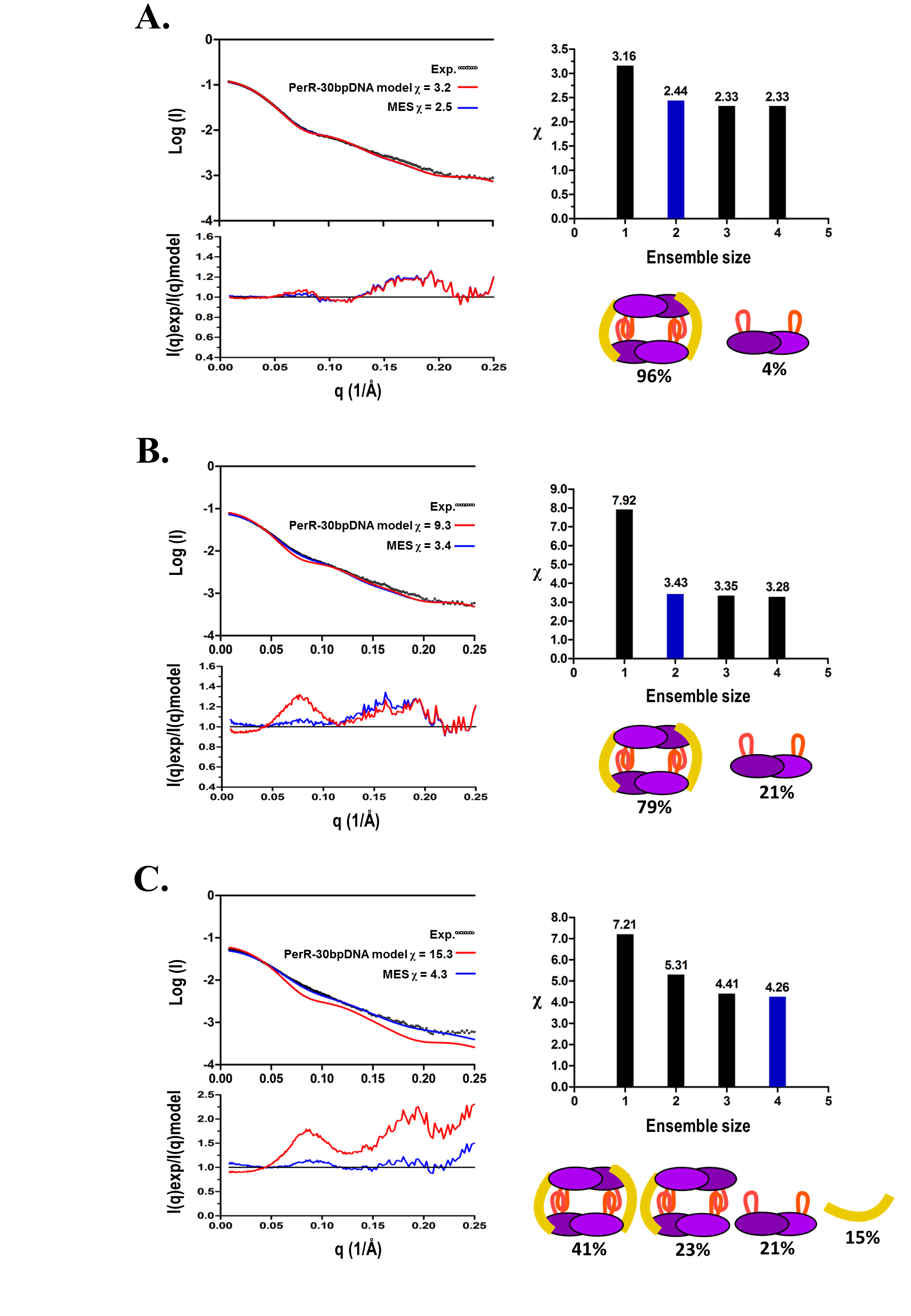

Supplement: Figure S5 — Minimal ensemble analysis of PerR-DNA complex mixtures. Ensemble fit (blue) versus PerR-30bpDNA model fit (red) by FoXS-MES for (A) 1∶1, (B) 2∶1, and (C) 4∶1, PerR:DNA ratio. Residual calculated as I(q)experimental/I(q)model is shown below the scattering curves. Chi value (χ) versus ensemble size is shown in histogram. The selected ensemble size from four input species (unbound DNA, protein, partial complex, complex) for each PerR:DNA ratio by MES is colored in blue in histogram and the selected species are represented in cartoon. (TIF) [file pone.0089027.s005.tif]
