# Supplementary material for: Distinct Structural Features of the Peroxide Response Regulator from Group A Streptococcus Drive DNA Binding
Source: PLoS One. 2014 Feb 21;9(2):e89027. doi: 10.1371/journal.pone.0089027 (PMC3931707; doi:10.1371/journal.pone.0089027)
Supplement: Table S1 — Primers for plasmids construction. (DOC) [file pone.0089027.s006.doc]

| **Primer Name** | **Forward Primer(5'→3')** | **Reverse Primer(5'→3')** |
| --- | --- | --- |
| PerR | GGGAATTCCATATGGACATTCATTCACATCAG | CCGCTCGAGAAAATCAGGTTGGTCTTTTG |
| PerR_H4A | ATGGACATT**GCT**TCACATCAGCAA | TTGCTGATGTGA**AGC**AATGTCCAT |
| PerR_H6A | GACATTCATTCA**GCT**CAGCAAGCCCTA | TAGGGCTTGCTG**AGC**TGAATGAATGTC |
| PerR_N15A | CTAGATGCCTATGAA**GCA**GTCCTAGAGCATCTAC | GTAGATGCTCTAGGAC**TGC**TTCATAGGCATCTAG |
| PerR_H19A | AATGTCCTAGAG**GCT**CTACGGGAAAAA | TTTTTCCCGTAG**AGC**CTCTAGGACATT |
| PerR_H97A | GACTTTATGGGC**GCT**CAACACGTCAAT | ATTGACGTGTTG**AGC**GCCCATAAAGTC |
| PerR_H99A | ATGGGCCATCAA**GCC**GTCAATGTAGTT | AACTACATTGAC**GGC**TTGATGGCCCAT |
| PerR_H44A | CAGTCCACAGAA**GCC**CCTAGTGCCGAT | ATCGGCACTAGG**GGC**TTCTGTGGACTG |
| PerR_N101A | CATCAACACGTC**GCA**GTAGTTTGTGAA | TTCACAAACTAC**TGC**GACGTGTTGATG |
| PerR_C104S | GTCAATGTAGTT**AGT**GAAATATGTGGG | CCCACATATTTC**ACT**AACTACATTGAC |
| PerR_C107S | GTAGTTTGTGAAATA**TCA**GGGAAGATTGCTGAC | GTCAGCAATCTTCCC**TGA**TATTTCACAAACTAC |
| PerR_C144S | attgcttacgggatt**tca**ccggattgccaagca | TGCTTGGCAATCCGG**TGA**AATCCCGTAAGCAAT |
| PerR_C147S | GGGATTTGTCCGGAT**TCA**CAAGCAAAAGAC | GTCTTTTGCTTG**TGA**ATCCGGACAAATCCC |
| PerR_R26A | AAACATATT**gCT**ATTACAgAgACACgT | CTCTgTAAT**AgC**AATATgTTTTTCCCg |
| PerR_R21A | gAgCATCTA**gCT**gAAAAACATATTCgA | ATgTTTTTC**AgC**TAgATgCTCTAggAC |
| PerR_R31A | ACAgAgACA**gCT**AAAgCCATTATTTCT | AATggCTTT**AgC**TgTCTCTgTAATTCg |
| PerR_N69A | gTCTATAAT**gCT**TTgAAAgTTTTAgTT | AACTTTCAA**AgC**ATTATAgACTgTggC |
| PerR_Y67A | GCCTTGCCACAGTC**GCT**AATAATTTGAAAG | CTTTCAAATTATT**AGC**GACTGTGGCAAGGC |
| PerR_N68A | CCTTGCCACAGTCTAT**GCT**AATTTGAAAGTTTTAG | CTAAAACTTTCAAATT**AGC**ATAGACTGTGGCAAGG |
| PerR_K71A | CAGTCTATAATAATTTG**GCA**GTTTTAGTTGATGAAGG | CCTTCATCAACTAAAAC**TGC**CAAATTATTATAGACTG |
| PerR_K83A | GTCTCAGAGTTG**GCA**ATCAGCAATG | CATTGCTGAT**TGC**CAACTCTGAGAC |

Table S1. Primers for plasmids construction
